# Patterns of energy availability and carbohydrate intake differentiate between adaptable and problematic low energy availability in female athletes

**DOI:** 10.3389/fspor.2024.1390558

**Published:** 2024-05-09

**Authors:** Birna Vardardottir, Sigridur Lara Gudmundsdottir, Ellen Alma Tryggvadottir, Anna S. Olafsdottir

**Affiliations:** Faculty of Health Promotion, Sport and Leisure Studies, University of Iceland, Reykjavik, Iceland

**Keywords:** sport nutrition, energy availability, relative energy deficiency, female athlete, nutrition status

## Abstract

**Background:**

Problematic low energy availability (EA) is the underlying culprit of relative energy deficiency in sport (REDs), and its consequences have been suggested to be exacerbated when accompanied by low carbohydrate (CHO) intakes.

**Objectives:**

This study compared dietary intake, nutrition status and occurrence of REDs symptoms in groups of female athletes, displaying different patterns of EA and CHO intake.

**Methods:**

Female athletes (*n* = 41, median age 20.4 years) from various sports weighed and recorded their food intake and training for 7 consecutive days via a photo-assisted mobile application. Participants were divided into four groups based on patterns of EA and CHO intakes: sufficient to optimal EA and sufficient to optimal CHO intake (SEA + SCHO), SEA and low CHO intake (SEA + LCHO), low energy availability and SCHO (LEA + SCHO), and LEA and LCHO (LEA + LCHO). SEA patterns were characterised by EA ≥30 and LEA by EA <30 kcal/kg fat free mass, and SCHO patterns characterised by CHO intake ≥3.0 and LCHO <3.0 g/kg body weight for most of the registered days. Body composition was measured with dual energy x-ray absorptiometry, resting metabolic rate with indirect calorimetry and serum blood samples were collected for evaluation of nutrition status. Behavioural risk factors and self-reported symptoms of REDs were assessed with the Low Energy Availability in Females Questionnaire, Eating Disorder Examination Questionnaire Short (EDE-QS), Exercise Addiction Inventory, and Muscle Dysmorphic Disorder Inventory.

**Results:**

In total, 36.6% were categorised as SEA + SCHO, of which 5/16 were ball sport, 7/10 endurance, 1/7 aesthetic, 2/5 weight-class, and 0/3 weight-class athletes. Of LEA + LCHO athletes (19.5% of all), 50% came from ball sports. Aesthetic and endurance athletes reported the greatest training demands, with weekly training hours higher for aesthetic compared to ball sports (13.1 ± 5.7 vs. 6.7 ± 3.4 h, *p* = 0.012). Two LEA + LCHO and one SEA + LCHO athlete exceeded the EDE-QS cutoff. LEA + LCHO evaluated their sleep and energy levels as worse, and both LEA groups rated their recovery as worse compared to SEA + SCHO.

**Conclusion:**

Repeated exposures to LEA and LCHO are associated with a cluster of negative implications in female athletes. In terms of nutrition strategies, sufficient EA and CHO intakes appear to be pivotal in preventing REDs.

## 1 Introduction

Energy availability (EA) refers to the residual dietary energy that is left for basic functions of the body after exercise energy expenditure (EEE) has been accounted for (1). Sufficient EA is fundamental for health and sport performance, while low EA (LEA) puts athletes at risk of relative energy deficiency in sport (REDs) (2–4). As outlined in the 2023 consensus update from the International Olympic Committee (IOC) (4), REDs is an umbrella term describing various health and performance decrements that may occur due to problematic LEA. Problematic LEA results from prolonged and/or severe exposure to LEA and disrupts function of one or more of the body systems. Exercise capacity, recovery, training adaptations, and other performance outcomes are consequently impaired. Conversely, adaptable LEA is short term and/or more benign LEA exposure, with little or no negative impact on health and performance. Whether a given scenario falls under adaptable or problematic LEA may be influenced by moderating factors, such as individual characteristics, athletes’ relationship with food and exercise, and nutrient composition of their diets (4, 5).

Early laboratory-based work in non-athletic females suggested that LEA, and resultant menstrual dysfunctions, occurred when EA went below 30 kcal/kg/fat free mass (FFM) (6, 7). Since then, this value has commonly been used as a cutoff for LEA. In recent years, the use of such a universal LEA cutoff has been debated for several reasons. That includes individual differences in endocrine and metabolic responses to LEA, and the distinction that has now been made between problematic and adaptable LEA (4, 8, 9). Although scientific understanding of REDs and its physiological consequences has vastly increased in the past decade, the degree of LEA (i.e., duration, magnitude and frequency) needed for it to be problematic remains unknown (4). However, recent intervention studies have found that as little as 1–2 weeks of exposure to LEA may be detrimental for health and performance outcomes in athletes across sports (10–12). Accumulating evidence also suggests that only a few consecutive days of insufficient or restricted carbohydrate (CHO) intake (<3.0 g/kg), with or without LEA, can impair physiological function and training adaptation (13–15). This is alarming given that athletes, and females especially, often fail to meet CHO requirements or intentionally restrict CHO intake (16–18). Also of note is that chronic CHO restriction often tends to modulate intakes of other important macro- and micronutrients (19). In contrast, nutrition periodisation often involves tailoring energy and/or CHO intake to training demands and/or stimulate fat oxidation capacity, or other training adaptations, by “training low” (e.g., in a fasted or CHO depleted state) (20, 21). This all comes down to the currently ill-defined threshold between beneficial versus harmful dietary modifications and behaviours in athletic populations.

Bearing the disparities between adaptable and problematic LEA in mind, not only average EA but also day-to-day variations deserve special consideration in real-life situations. Such variations or patterns have been described in a few small (*n* < 15) single sport and case studies (22–25) but not in larger cross-sectional investigations. Therefore, the aim of the present study was to compare dietary intake, nutrition status and occurrence of REDs symptoms, between groups displaying different patterns of EA and CHO intake.

## 2 Methods

### 2.1 Study population

This study used data from a larger cross-sectional research project on REDs in high-level Icelandic athletes aged ≥15 years. The eligibility criteria and recruitment of participants have been described in detail elsewhere (26). The athletes initially responded to an online questionnaire in 2021 (July–December) consisting of the Low Energy Availability in Females Questionnaire (LEAF-Q) (27) and additional background questions. The measurement phase was between April and September 2022. Of the 56 female athletes that participated in the measurement phase, 48 logged their dietary intake and training via a photo-assisted mobile application. Seven participants provided insufficient registration (<5 days dietary intake recorded and/or no training session registered) for determination of EA. Therefore, 41 athletes were included in the present analysis. For most participants (*n* = 35), 7 days of registered dietary intake and training were available. Participants with 6 (*n* = 5) or 5 (*n* = 1) days were also included. The athletes came from five different sport groups, using definitions suggested by another study (28): ball (39%); endurance (24.4%); aesthetic (17.1%); weight-class (12.2%); and power sports (7.3%).

### 2.2 Energy availability

#### 2.2.1 Digital food and training records

Participants recorded their dietary intake and training for 7 consecutive days. Weighed amounts and descriptions of all foods eaten in addition to photographs of foods taken/served as well as leftovers were registered via a mobile application (app). The app is in Icelandic and was originally developed for a study on eating behaviours in children and their parents (29) but was subsequently adapted to fit the needs of the present study, mainly by adding a training record and a more detailed logging option for exact amounts of foods consumed. Participants received individual encoded login information and detailed instructions on how to use the app. All were verbally informed about the aims of this registration, and the importance of not changing what, when and how much they ate because of their participation in the study.

**Figure 1:**
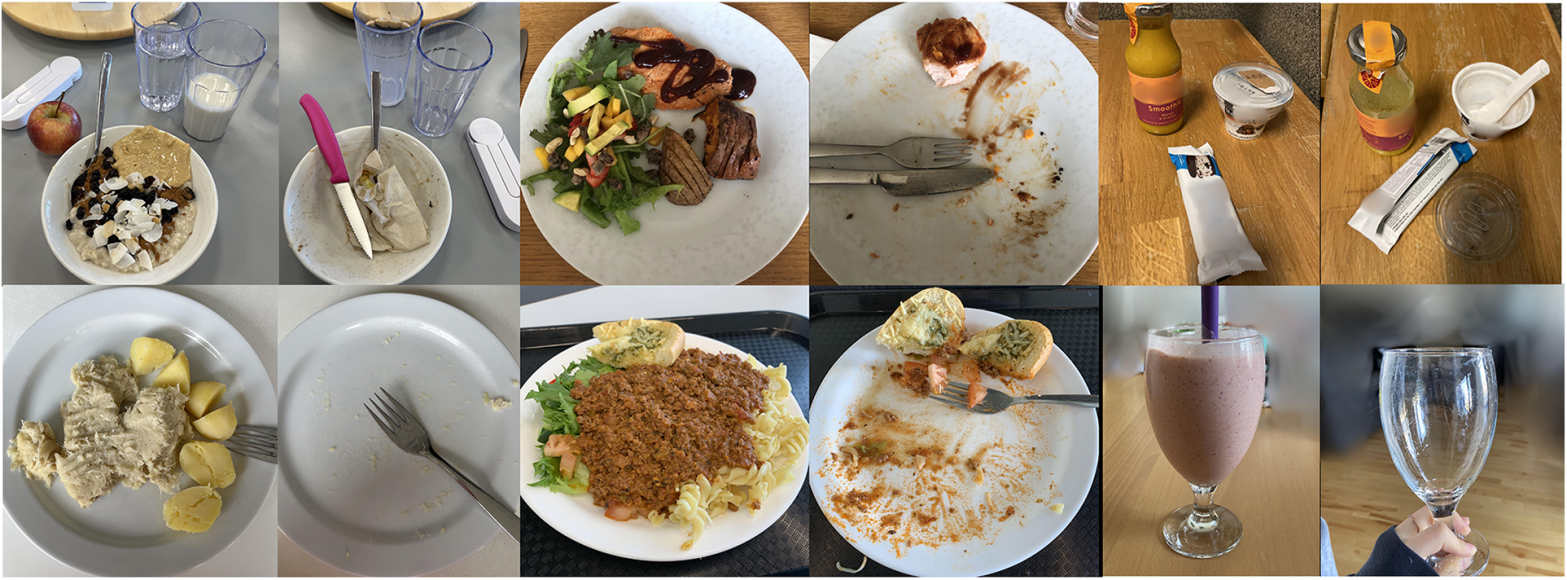
Examples of before and after photos of meals from the food records. The examples are random and from six different individuals.

The athletes logged all food, drinks other than still water, dietary supplements, and ergogenic aids. Similar to the remote food photography method used in a previous study on athletes (22), before and after photos of meals and snacks were taken (directly in the app or uploaded from the photo gallery) and kitchen scales (provided if needed) were used to weigh each food/meal item. Assessment of dietary intakes from the photographs was based on a validated method (30). In cases where food weighing and/or photographing was not possible, participants were asked to provide written information, such as a description of what was ordered from restaurant menus. They also had the option to include additional information or photos, e.g., of ingredient lists or labels on food packaging. The use of sport foods and supplements was manually derived from the app registrations, using descriptions provided by the IOC (31). The app did not provide any calculations or feedback to participants, but they could see an overview of meals and meal timings they had logged. Examples of before and after photos from meals are provided in Figure 1.

During the same period, participants reported training sessions, their duration and rated perceived exertion based on the Borg rating scale in the app (32). If a training watch or global positioning system (GPS) device was used, participants were asked to register the highest heart rate reached during the session and type of measurement (e.g., watch/wrist or chest strap). Lastly, the participants were asked to write a short description of the training session, where they could also add a photo of their training plan/workout description.

#### 2.2.2 Body composition and resting metabolic rate

Body composition, including FFM, was measured in a rested and fasted state via whole-body dual energy x-ray absorptiometry (DXA). The FFM index (FFMI) was calculated by dividing the total FFM (kg) by height squared (m^2^) (33). Most participants (*n* = 36) had a valid assessment of fasted resting metabolic rate (RMR), conducted via indirect calorimetry (ventilated hood). For those, the RMR ratio was calculated by dividing the measured value with the estimated value from the Cunningham formula (34). RMR ratio <0.90 is among suggested secondary markers of REDs (35, 36). DXA and RMR measurement procedures have been described in detail elsewhere (26).

#### 2.2.3 Energy availability and nutrient intake calculations

All food records were coded by the same nutritionist. The data were then transported into the ICEFOOD calculation programme, which is based on the Icelandic food composition database (ISGEM), for energy and nutrient intake calculations. ICEFOOD was initially developed for the national diet survey in Iceland in 2002 and has also been used for more recent surveys as well as for research purposes (37, 38). As part of the data cleansing process, coding and calculations were thoroughly checked and any evident errors corrected by the research team. In accordance with Icelandic and Nordic nutrition recommendations (39, 40), low intakes of micronutrients were defined as vitamin D <15 µg, iron <15 mg, folate <300 µg, and vitamin B12 <2 µg.

Registered training hours and number of sessions were derived from the training records. Weekly training hours and sessions for athletes who registered 5 or 6 days in the app were calculated by dividing the number of training hours/sessions by number of registered days and then multiplied by seven. For comparison, athletes were asked how many hours (on average) they usually trained per week, before starting the registration via questionnaire. EEE was estimated from the training logs based on reported exercise mode, intensity or perceived exertion, and duration, as described by Heikura et al. (41). Each training session, or parts of it, was assigned a relevant metabolic equivalent (MET) value for the type and intensity of the activity (42). MET scores were then multiplied by the session/activity duration for the calculation of total EEE. RMR, either measured (if valid measures were available) or estimated via the Cunningham formula (34), was subtracted from total EEE to yield energy cost of the workout alone.

Daily EA was calculated using the following formula (1):

$$\mathrm{Energy availability}=\frac{\mathrm{Energy intake}\;(\mathrm{kcal})\;-\;\mathrm{Exercise energy expenditure}\;(\mathrm{kcal})}{\mathrm{Fat free mass}\;(\mathrm{kg})}$$

#### 2.2.4 Categorisation based on energy availability and carbohydrate intake patterns

Based on a graphical presentation of individual day-to-day patterns of EA and nutrient intake [Supplementary Information (SI)], participants were manually divided into four groups based on patterns of EA and CHO intake:

1. SEA + SCHO: sufficient to optimal energy availability + sufficient to optimal carbohydrate intake.
2. SEA + LCHO: sufficient to optimal energy availability + low carbohydrate intake.
3. LEA + SCHO: low energy availability + sufficient to optimal carbohydrate intake.
4. LEA + LCHO: low energy availability + low carbohydrate intake.

SEA patterns were characterised by EA ≥30 kcal/kg and LEA by EA <30 kcal/kg FFM for most days (≥5 out of 7 or ≥4 out of 5–6 days). SCHO patterns were characterised by CHO intake ≥3.0 and LCHO <3.0 g/kg for most days (≥4 out of 7 or ≥3 out of 5–6 days). The categorisation was further confirmed by calculated averages, where the two LEA groups had an average EA <30 kcal/kg FFM/day and those with sufficient to optimal EA averaged ≥30 kcal/kg FFM/day. Likewise, the groups with LCHO had an average CHO intake <3.0 g/kg/day and the SCHO groups averaged ≥3.0 g/kg/day.

### 2.3 Serum nutrition status

Fasted serum blood samples were collected and analysed as described earlier (26). Ferritin, iron, and total iron binding capacity (TIBC) were measured for the evaluation of iron status, and 25-hydroxyvitamin D (25-OH-Vitamin D) for vitamin D status. Using the laboratory reference values, low ferritin (adolescents <12 µg/L, adults <15 µg/L), low iron (<10 µmol/L), and high TIBC (>70 µmol/L) served as indicators of iron deficiency. In addition, transferrin saturation (TSAT) was calculated using the following formula: (iron/TIBC) × 100, with TSAT <20% considered low (43). Vitamin D insufficiency is defined as serum 25-OH-Vitamin D concentrations <50 nmol/L, and concentrations <30 nmol/L are a marker of vitamin D deficiency (40). The prevalence of concentrations below 80 nmol/L, an often-used definition of insufficiency in athletes (3, 44), was also evaluated. Other measured markers of nutrition status were calcium (reference range: 2.15–2.6 mmol/L), magnesium (reference range: 0.74–0.99 mmol/L), and vitamin B12 (reference range: 142–725 pmol/L).

### 2.4 Self-reported symptoms of LEA

#### 2.4.1 Low Energy Availability Questionnaire

The Low Energy Availability in Females Questionnaire (LEAF-Q) was used to screen for physiological symptoms of REDs. Total score of ≥8, injury sub-score ≥2, gastrointestinal (GI) sub-score ≥2, and menstrual sub-score ≥4 are the established cutoffs for LEAF-Q (27).

LEAF-Q also assesses menstrual disturbances (oligomenorrhea or amenorrhoea) and use of contraceptives. Athletes with <9 menses in the past 12 months, in absence of hormonal contraceptive use, were defined as having menstrual disturbances, and those currently using hormonal contraceptives were defined as contraceptive users (45). Menstrual function was not defined for one athlete due to perimenopausal age (>45 years).

LEAF-Q was supplemented by questions retrieved from the recently developed Low Energy Availability in Males Questionnaire (LEAM-Q) (46). The LEAM-Q-derived categories concerned dizziness, thermoregulation at rest, fatigue (lethargy, tiredness, lack of concentration in general), fitness (body pain, muscle stiffness, physical exhaustion, and vulnerability to injuries), sleep, recovery (physical recovery and perceived training progress), and energy levels (training readiness, perceived happiness, and energetic levels). Scores for LEAM-Q-derived measures were calculated according to the initial scoring key. Validated LEAM-Q cutoffs, other than for male-specific sex drive, are currently lacking but higher scores indicate a worse outcome (46).

#### 2.4.2 Disordered eating, compulsive exercise, and muscle dysmorphia

All participants responded to the Eating Disorder Examination—Questionnaire Short (EDE-QS) (47), Exercise Addiction Inventory (EAI) (48), and Muscle Dysmorphic Disorder Inventory (MDDI) (49). The established cutoffs are ≥15 for EDE-QS and ≥39 for MDDI. An EAI score ≥24 indicates a risk of compulsive exercise, 13–23 some symptoms, and 6–12 no symptoms.

### 2.5 Data analysis

Statistical analyses were conducted using IBM SPSS statistics 29.0.1.1, with significance set to *α*<0.05. Distributions of all data were checked using the Shapiro–Wilk test and visual inspection of Q-Q plots. Continuous variables were summarised as mean ± SD for normally distributed data, and medians with 25th and 75th interquartile ranges (IQR) for non-parametric data. Cross-tabulation and Pearson chi-square statistics were used for the evaluation of categorical data, including the occurrence of LEAF-Q, EDE-QS, EAI, and MDDI scores above cutoff.

Participant training characteristics, dietary intakes, and questionnaire scores were compared with one-way analysis of variance (ANOVA) and Kruskal–Wallis statistics. The independent samples *t*-test was used to compare differences in nutrient intake between athletes using sport foods and supplements compared to non-users. Body composition and nutrition status were compared based on EA + CHO categorisation (fixed factor) and adjusted for age using analysis of covariance (ANCOVA). When appropriate, Bonferroni *post hoc* for multiple comparisons was applied. For pairwise comparisons, mean differences (MD) and confidence intervals (95% CI) were reported for parametric data, and effect size (*r* = Z/√n) for non-parametric outcomes with threshold values set at 0.1 (small effects), 0.3 (moderate), and 0.5 (large) (50).

## 3 Results

### 3.1 Participant characteristics

Participant characteristics, with age adjusted comparisons, are summarised in Table 1. The age range was 15–48 years but did not differ between the EA + CHO groups (Kruskal–Wallis H = 1.227, *p* = 0.747). The SEA + LCHO group had a higher body weight (BW) compared to the LEA + SCHO [MD = 14.0 kg (2.2–25.8), *p* = 0.013] and SEA + SCHO groups [MD = 16.4 kg (5.7–27.1), *p* < 0.001]. Group differences were observed for FFM and FFMI and *post hoc* analyses revealed that the SEA + LCHO group had a higher FFMI compared to the SEA + SCHO group [MD = 1.2 kg/m^2^ (0.06–2.4), *p* = 0.034]. The SEA + LCHO group also had the highest body fat percentage and differed significantly from the LEA + SCHO [MD = 10.1% (2.4–17.8), *p* = 0.005] and SEA + SCHO groups [MD = 9.2% (2.2–16.2), *p* = 0.005] but not from the LEA + LCHO group. No group differences were observed for whole-body bone mineral density (BMD) Z scores and RMR. One athlete, in the SEA + SCHO group, had a RMR ratio <0.90. Of the athletes aged <45 years, 30% were using hormonal contraceptives, either oral contraceptive pills (*n* = 9) or other forms: ring, coil, and injections (*n* = 3). Previous (i.e., not current) use of oral contraceptives was reported by 10 (25%) athletes. Current amenorrhoea was reported by one athlete, and that athlete was categorised as LEA + LCHO and not taking contraceptives. A considerably larger proportion (*n* = 13) had a history of amenorrhoea and six reported current oligomenorrhea.

**Table 1:** Participant characteristics.

		EA + CHO groups				Age adjusted ANCOVA		
	All *n* = 41	SEA + SCHO *n* = 15	SEA + LCHO *n* = 9	LEA + SCHO *n* = 9	LEA + LCHO *n* = 8	F	*p*-value	ηp^2^
Age	20.4 (17.9–27.0)	22.3 (16.7–31.5)	20.3 (17.7–23.6)	19.8 (17.7–25.0)	22.2 (20.1–31.3)	—	—	—
Body weight (kg)	65.7 ± 10.8	59.7 ± 9.3	75.6 ± 12.5	61.7 ± 5.4	70.4 ± 5.6	7.406	<0.001	0.382
BMI (kg/m^2^)	22.9 ± 3.3	21.4 ± 2.8	26.1 ± 3.5	21.5 ± 2.0	23.6 ± 2.6	6.814	<0.001	0.362
DXA FFM (kg)	45.5 ± 4.6	43.0 ± 4.0	47.6 ± 4.7	45.1 ± 2.6	48.4 ± 5.0	4.313	0.011	0.264
DXA FFMI (kg/m^2^)	15.9 ± 1.1	15.4 ± 1.2	16.5 ± 0.9	15.7 ± 1.0	16.1 ± 0.9	3.003	0.043	0.200
DXA fat mass (kg)	17.4 ± 7.3	14.1 ± 6.0	24.8 ± 8.2	13.8 ± 3.1	19.1 ± 5.5	7.058	<0.001	0.370
DXA fat%	25.7 ± 6.8	23.0 ± 6.4	32.4 ± 5.6	22.3 ± 3.3	27.0 ± 6.8	6.010	0.002	0.334
DXA whole-body BMD Z-score	1.3 ± 1.0	1.1 ± 1.1	1.1 ± 1.1	1.7 ± 1.0	1.4 ± 0.9	1.035	0.389	0.079
RMR (kcal)^a^	1,637 ± 217	1,591 ± 227	1,702 ± 224	1,600 ± 266	1,691 ± 149	0.876	0.464	0.078
RMR/FFM (kcal/kg FFM)^a^	35.8 ± 3.8	36.5 ± 3.6	35.8 ± 2.7	35.1 ± 5.3	35.2 ± 4.1	0.224	0.879	0.021
RMR ratio^a,b^	1.08 ± 0.11	1.09 ± 0.11	1.10 ± 0.09	1.06 ± 0.17	1.08 ± 0.10	0.141	0.935	0.013
	*n* = 40	*n* = 14	*n* = 9	*n* = 9	*n* = 8			
Menstrual function								
Normal menstruation *n* (%)	21 (52.5)	7 (50.0)	3 (33.3)	8 (88.9)	3 (37.5)			
Menstrual disturbances *n* (%)	7 (17.5)	4 (28.6)	1 (11.1)	0	2 (25.0)			
Contraceptive use *n* (%)	12 (30.0)	3 (21.4)	5 (55.6)	1 (11.1)	3 (37.5)			

Categorical data are presented as *n* and within-group frequencies (%), parametric continuous variables as means ± SD and nonparametric as median (25p–75p interquartile range). BMI, body mass index; DXA, dual energy X-ray absorptiometry; FFM, fat free mass; FFMI, FFM index (total FFM/height squared); BMD, bone mineral density. Energy availability (EA) and carbohydrate (CHO) groups = SEA + SCHO, sufficient to optimal EA and sufficient to optimal CHO intake; SEA + LCHO, sufficient to optimal EA and low CHO intake; LEA + SCHO, low EA and sufficient to optimal CHO; LEA + LCHO, low EA and low CHO intake. Menstrual function was defined based on responses to the Low Energy Availability in Females Questionnaire (LEAF-Q).

^a^ RMR measured via indirect calorimetry (valid measure available for 36 participants).

^b^ Calculated RMR ratio: measured RMR/estimated via the Cunningham formula.

EA + CHO categorisation and training characteristics of the five sports groups are shown in Table 2. Most participants were categorised as SEA + SCHO (36.6%), of whom 70% were the endurance and 31.3% were the ball sport athletes. Moreover, half (*n* = 8/16) of the ball sport athletes had patterns characterised by LEA and that was accompanied by LCHO in four of them. Four out of seven aesthetic athletes but none of the weight-class athletes had LEA patterns.

**Table 2:** EA + CHO categorisation, weekly training load, and EEE.

Variable	Ball *n* = 16	Endurance *n* = 10	Aesthetic *n* = 7	Weight-class *n* = 5	Power *n* = 3	F/H^a^	*p*-value
EA + CHO groups						—	—
SEA + SCHO *n* (%)	5 (31.3)	7 (70.0)	1 (14.3)	2 (40.0)	0		
SEA + LCHO *n* (%)	3 (18.8)	0	2 (28.6)	3 (60.0)	1 (33.3)		
LEA + SCHO *n* (%)	4 (25.0)	2 (20.0)	3 (42.9)	0	0		
LEA + LCHO *n* (%)	4 (25.0)	1 (10.0)	1 (14.3)	0	2 (66.7)		
Weekly training sessions (min–max)	5.6 ± 2.3 (2–10)	7.9 ± 2.4 (5–12)	8.0 ± 4.2 (3–14)	4.8 ± 1.6 (2–7)	5.7 ± 2.3 (3–7)	2.195	0.089
Training hours/week							
From questionnaire	9.7 ± 3.2	12.4 ± 3.6	15.7 ± 4.6	9.3 ± 2.2	11.2 ± 1.6	4.368	0.006
From training diary	6.7 ± 3.4	10.6 ± 3.7	13.1 ± 5.7	6.3 ± 3.1	8.7 ± 4.7	4.116	0.008
Average daily EEE							
kcal	322 (210–564)	473 (372–710)	595 (351–758)	286 (153–365)	374 (258–)	11.425	0.022
kcal/kg FFM	6.6 (4.9–12.0)	9.7 (8.6–16.6)	12.6 (8.0–17.0)	5.9 (4.0–7.9)	7.3 (5.7–)	12.579	0.014

Categorical data are presented as *n* and within-sport group frequencies (%), parametric continuous variables as means ± SD and nonparametric as median (25p–75p interquartile range). Ball: Football, handball, basketball, volleyball, badminton; Endurance: middle to long distance running, swimming, cycling; Aesthetic: gymnastics, figure skating, ballroom dancing, ballet; Weight-class: wrestling, powerlifting, karate; Power: sprinting, throwing, and jumping events, alpine skiing. FFM, fat free mass; EEE, exercise energy expenditure. Energy availability (EA) and carbohydrate (CHO) groups = SEA + SCHO, sufficient to optimal EA and sufficient to optimal CHO intake; SEA + LCHO, sufficient to optimal EA and low CHO intake; LEA + SCHO, low EA and sufficient to optimal CHO; LEA + LCHO, low EA and low CHO intake.

^a^ ANOVA (*F* values) and Kruskal Wallis (H-Values) group comparisons.

Between-sport group differences were observed for weekly number of training hours and daily EEE. Multiple comparisons were not significant for EEE after Bonferroni corrections were applied. However, average training hours from the training records were higher in aesthetic athletes than ball sport athletes [MD = 6.4 h (0.95–11.8), *p* = 0.012]. The weekly number of training hours, derived from the questionnaire, was also higher in aesthetic sports compared to ball [MD = 6.0 h (1.3–10.7), *p* = 0.005] and weight-class [MD = 6.4 h (0.4–12.4), *p* = 0.030] sports.

### 3.2 Energy availability and dietary intake

In addition to differences in CHO intake, intakes of protein, fat, and fibre differed by groups (Table 3). More specifically, the LEA + LCHO group had the lowest relative intake of all macronutrients and the SEA + SCHO group had the highest. The average protein intake differed between SEA + SCHO and LEA + LCHO [MD = 0.6 g/kg (0.04–1.1), *p* = 0.028]. SEA + SCHO also had higher fat intakes compared to SEA + LCHO [MD = 0.4 g/kg (0.1–0.7), *p* = 0.004] and LEA + LCHO [MD = 0.6 g/kg (0.3–0.9), *p* < 0.001]. Finally, fibre intake was higher in SEA + SCHO compared to LEA + LCHO [MD = 0.2 g/kg (0.02–0.47), *p* = 0.028].

**Table 3:** Seven-day average energy availability and dietary intakes, with one-way ANOVA comparisons between EA + CHO groups.

		EA + CHO groups				ANOVA		
	All *n* = 41	SEA + SCHO *n* = 15	SEA + LCHO *n* = 9	LEA + SCHO *n* = 9	LEA + LCHO *n* = 8	F	*p*-value	ηp^2^
Energy availability (kcal/kg FFM)	35.5 ± 10.0	45.1 ± 6.5	36.1 ± 5.8	29.6 ± 2.5	23.3 ± 5.5	31.354	<0.001	0.718
Energy intake								
kcal	2,043 ± 362	2,341 ± 303	2,001 ± 335	1,926 ± 178	1,661 ± 145	12.593	<0.001	0.505
kcal/kg	31.9 ± 7.7	39.7 ± 5.4	26.7 ± 4.3	31.4 ± 4.0	23.6 ± 1.8	29.660	<0.001	0.706
kcal from sport foods/supplements	142 ± 126	157 ± 139	191 ± 169	86 ± 67	121 ± 79	1.210	0.320	0.089
Carbohydrate intake								
g	212 ± 54	257 ± 55	187 ± 36	207 ± 14	161 ± 24	11.905	<0.001	0.491
g/kg	3.3 ± 1.1	4.3 ± 0.9	2.5 ± 0.4	3.4 ± 0.4	2.3 ± 0.4	26.668	<0.001	0.684
g from sport foods/supplements	10.8 ± 9.9	12.1 ± 11.1	14.9 ± 11.9	6.0 ± 6.0	9.2 ± 7.4	1.386	0.262	0.101
Protein intake								
g	106 ± 25	112 ± 26	115 ± 28	95 ± 14	96 ± 22	1.930	0.142	0.135
g/kg	1.6 ± 0.5	1.9 ± 0.6	1.6 ± 0.4	1.5 ± 0.2	1.4 ± 0.3	3.631	0.022	0.227
g from sport foods/supplements	15.7 ± 15.4	15.2 ± 16.2	22.7 ± 20.2	11.4 ± 10.5	13.8 ± 12.1	0.887	0.457	0.067
Fat intake								
g	81 ± 18	90 ± 20	84 ± 18	75 ± 12	67 ± 10	4.059	0.014	0.248
g/kg	1.3 ± 0.3	1.5 ± 0.3	1.1 ± 0.2	1.2 ± 0.2	1.0 ± 0.1	9.882	<0.001	0.445
g from sport foods/supplements	3.7 ± 3.9	4.9 ± 4.9	4.4 ± 4.5	1.8 ± 1.6	2.5 ± 1.4	1.617	0.202	0.116
Fibre intake								
g	21.1 ± 10.0	26.4 ± 13.6	18.5 ± 5.6	20.5 ± 6.2	14.7 ± 3.4	3.127	0.037	0.202
g/kg	0.3 ± 0.2	0.5 ± 0.3	0.3 ± 0.1	0.3 ± 0.1	0.2 ± 0.1	3.895	0.016	0.240
g from sport foods/supplements	1.0 ± 1.8	1.6 ± 2.4	0.7 ± 1.7	0.3 ± 0.5	1.0 ± 1.6	0.994	0.406	0.075
Vitamin D intake (µg)								
Total	9.3 ± 9.1	10.9 ± 11.5	11.7 ± 10.2	7.5 ± 6.2	5.7 ± 3.3	0.903	0.449	0.068
From supplements	4.4 ± 8.7	5.8 ± 11.4	6.4 ± 9.7	2.4 ± 5.7	1.6 ± 3.1	0.721	0.546	0.055
Iron intake (mg)	12.9 ± 4.5	14.3 ± 3.9	11.2 ± 2.9	14.4 ± 6.7	10.4 ± 2.1	2.326	0.091	0.159
Folate intake (µg)	350 ± 138	386 ± 108	313 ± 158	387 ± 165	278 ± 116	1.578	0.211	0.113
Vitamin B12 intake (µg)	6.2 ± 3.7	7.0 ± 4.1	6.9 ± 5.2	4.8 ± 1.2	5.1 ± 2.0	1.039	0.387	0.078

Data are presented as mean ± SD.

Energy availability (EA) and carbohydrate (CHO) groups: SEA + SCHO, sufficient to optimal EA and sufficient to optimal CHO intake; SEA + LCHO, sufficient to optimal EA and low CHO intake; LEA + SCHO, low EA and sufficient to optimal CHO; LEA + LCHO, low EA and low CHO intake.

A total of 36 (87.8%) athletes used sport foods and/or supplements, of whom, two used vitamins and minerals only. Vitamin D intake was <15 µg in 82.9% of all athletes but 12 (29.3%) took supplements with vitamin D and their total intake averaged at 19.5 µg compared to 5.1 µg in those who did not supplement. Vitamins without D were taken by 5 (12.2%) and minerals or electrolytes by 11 (26.8%). Iron intake was <15 mg in 78% of all athletes, but supplements with iron were taken by 4 (9.8%). Folate intake was <300 µg in 46.3% of all. None had low intakes of vitamin B12.

In total, 32 (78%) athletes used protein supplements and/or protein-enriched products. Of them, 27 (65.9%) used protein-enriched dairy or ready to serve protein drinks, 13 (31.7%) used protein powders, and 21 (51.2%) used protein bars or snacks. The average protein intake of those who used protein products was 1.7 g/kg (range 1.0–3.1) compared to 1.3 g/kg (range 0.8–1.8) for those who did not (*p* = 0.019). The use of energy drinks and/or pre-workout products was reported by 14 (34.1%) athletes, exogenous CHO, such as sport drinks, gels, and bars, by 10 (24.4%) and ergogenic aids such as creatine by 4 (9.8%). The contribution of sport foods and supplements to the total protein intake ranged between 12% in the LEA + SCHO group and 19.7% in the SEA + LCHO group. Similarly, sport foods and supplements contributed 2.9 (LEA + SCHO) to 8.0% (SEA + LCHO) to the total CHO intake.

### 3.3 Serum nutrition status

Between-group differences were not observed for nutrition status (Table 4). None of the SEA + SCHO athletes had vitamin D deficiency or insufficiency, but one in the LEA + LCHO group was deficient (<30 nmol/L) and three were insufficient (<50 nmol/L). Moreover, three athletes in the LEA + SCHO group and two in the SEA + LCHO group were deficient in vitamin D. Of all participants, 31 (75.6%) had vitamin D concentrations below the frequently used cutoff for insufficiency in athletes, i.e., 80 nmol/L. No apparent differences were found in vitamin D status between those who used vitamin D supplements and those who did not (66.7 ± 21.7 vs. 65.3 ± 16.9 nmol/L, *p* = 0.838). Ferritin was below reference for age in five (12.2%) participants, and was accompanied by low iron and/or high TIBC in four. In addition, six athletes had low levels of iron only, of which four were normally menstruating. Of those five with low ferritin, three were normally menstruating. TSAT was <20% in 10 athletes, with no apparent group differences. No athlete was deficient in vitamin B12, magnesium, and calcium.

**Table 4:** Serum nutrition status.

Dependent variables	EA + CHO groups					Age adjusted ANCOVA		
	ALL *n* = 41	SEA + SCHO *n* = 15	SEA + LCHO *n* = 9	LEA + SCHO *n* = 9	LEA + LCHO *n* = 8	F	*p*-value	ηp^2^
25-OH-Vitamin D (nmol/L)	66.3 ± 20.2	70.1 ± 12.9	68.7 ± 25.3	66.2 ± 20.1	56.5 ± 25.7	0.802	0.501	0.063
Vitamin B12 (pmol/L)	481 ± 197	460 ± 187	505 ± 267	505 ± 209	468 ± 131	0.250	0.861	0.020
Fe (µmol/L)	17.1 ± 7.6	18.1 ± 7.7	16.4 ± 9.1	16.3 ± 9.1	16.9 ± 4.7	0.058	0.981	0.005
Ferritin (µg/L)	50.4 ± 32.8	54.5 ± 29.7	54.4 ± 39.5	42.6 ± 35.9	46.9 ± 31.0	0.286	0.835	0.023
TIBC (µmol/L)	59.9 ± 9.4	56.9 ± 8.0	58.8 ± 9.4	64.7 ± 11.5	61.1 ± 8.4	1.318	0.284	0.102
Transferrin saturation (%)	29.9 ± 14.2	32.6 ± 13.8	29.2 ± 18.9	27.2 ± 15.6	28.2 ± 9.2	0.212	0.887	0.018
Calcium (mmol/L)	2.36 ± 0.07	2.35 ± 0.79	2.34 ± 0.06	2.40 ± 0.06	2.34 ± 0.04	1.345	0.275	0.101
Magnesium (mmol/L)	0.84 ± 0.04	0.84 ± 0.05	0.84 ± 0.04	0.84 ± 0.03	0.83 ± 0.05	0.219	0.882	0.018

Fe, iron; TIBC, total iron binding capacity. Energy availability (EA) and carbohydrate (CHO) groups = SEA + SCHO, sufficient to optimal EA and sufficient to optimal CHO intake; SEA + LCHO, sufficient to optimal EA and low CHO intake; LEA + SCHO, low EA and sufficient to optimal CHO; LEA + LCHO, low EA and low CHO intake. Data presented as mean ± SD for each EA + CHO group.

### 3.4 Self-reported symptoms of LEA

#### 3.4.1 Low Energy Availability Questionnaire

The median LEAF-Q total score for all athletes was 6 (IQR: 4–10) with 41.5% scoring above the cutoff (≥8). Moreover, 58.5% scored above the injury (≥2), 43.9% above the gastrointestinal cutoff (≥2) and 26.8% above the menstrual score (≥4) cutoffs, with no apparent differences between EA + CHO groups (Pearson's chi-square *p* > 0.05 for all). All 7 athletes with menstrual disturbances scored above the menstrual cutoff, and so did 2 out of the 12 contraceptive users.

ANOVA and the Kruskal–Wallis test showed the differences between EA + CHO groups in calculated LEAM-Q-derived sleep, recovery, and energy levels scores (Table 5). Pairwise Bonferroni *post-hoc* tests revealed that LEA + LCHO had higher median sleep scores (*r* = 0.50, *p* = 0.007) and higher mean energy level scores [MD = 3.2 (0.5–5.9), *p* = 0.013] compared to SEA + SCHO. Both LEA + LCHO and LEA + SCHO had higher recovery scores compared to SEA + SCHO (*r* = 0.43, *p* = 0.030; and *r* = 0.51, *p* = 0.007, respectively).

**Table 5:** Between-group comparison of LEAF-Q- and LEAM-Q-derived scores.

	EA + CHO groups				Kruskal–Wallis/ANOVA	
Questionnaire measures	SEA + SCHO *n* = 15	SEA + LCHO *n* = 9	LEA + SCHO *n* = 9	LEA + LCHO *n* = 8	H	*p*-value
LEAF-Q total	6.0 (3.0–12.0)	7.0 (3.0–9.5)	5.0 (3.0–8.0)	9.0 (6.0–14.3)	4.650	0.199
LEAF-Q injury	0.0 (0.0–5.0)	2.0 (0.0–3.5)	2.0 (0.0–4.0)	2.5 (0.5–3.8)	0.476	0.924
LEAF-Q gastro-intestinal	2.0 (1.0–3.0)	2.0 (1.0–3.0)	1.0 (1.0–2.0)	3.0 (1.5–5.5)	6.866	0.076
LEAF-Q menstrual	2.5 (0.0–4.0)	2.0 (0.5–3.0)	1.0 (0.5–3.0)	3.0 (0.5–7.8)	1.970	0.579
Dizziness^a^	1.0 (0.0–3.0)	2.0 (0.5–3.0)	1.0 (0.5–3.5)	2.0 (1.3–3.0)	3.488	0.322
Thermoregulation^a^	1.0 (0.0–2.0)	1.0 (0.0–2.0)	2.0 (1.0–4.5)	3.5 (1.3–5.8)	7.829	0.050
Sleep^a^	1.0 (1.0–4.0)	5.0 (1.0–6.0)	5.0 (2.0–7.5)	6.5 (3.3–10.8)	12.012	0.007
Recovery^a^	2.0 (1.0–4.0)	4.0 (1.5–5.0)	5.0 (4.0–7.0)	5.0 (4.0–5.0)	13.830	0.003
					F	*p*-value
Fatigue^a^	3.8 ± 2.6	6.2 ± 4.3	6.2 ± 3.9	7.9 ± 3.4	2.687	0.060
Fitness^a^	4.7 ± 3.3	5.8 ± 3.0	6.9 ± 5.0	7.9 ± 3.2	1.548	0.218
Energy levels^a^	1.9 ± 2.0	4.2 ± 1.7	4.3 ± 3.2	5.1 ± 1.8	4.600	0.008

Energy availability (EA) and carbohydrate (CHO) groups = SEA + SCHO, sufficient to optimal EA and sufficient to optimal CHO intake; SEA + LCHO, sufficient to optimal EA and low CHO intake; LEA + SCHO, low EA and sufficient to optimal CHO; LEA + LCHO, low EA and low CHO intake.

^a^ Data presented as median (interquartile range, p25–p75) for nonparametric and mean ± SD for parametric measures. LEAF-Q, Low Energy Availability in Females Questionnaire; scores derived from LEAM-Q, Low Energy Availability in Males Questionnaire.

#### 3.4.2 Disordered eating, compulsive exercise, and muscle dysmorphia

**Figure 2:**
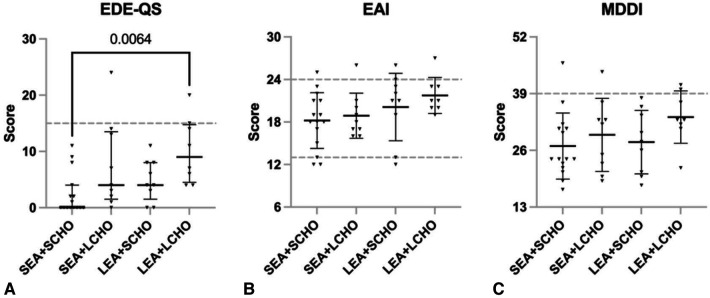
Scores on (**A**) Eating Disorder Examination - Questionnaire Short (EDE-QS, median with 25p–75p interquartile range), (**B**) Exercise Addiction Inventory (EAI, mean ± SD), and (**C**) Muscle Dysmorphic Disorder Inventory (MDDI, mean ± SD) for the four energy availability (EA) and carbohydrate (CHO) groups = SEA + SCHO, sufficient to optimal EA and sufficient to optimal CHO intake; SEA + LCHO, sufficient to optimal EA and low CHO intake; LEA + SCHO, low EA and sufficient to optimal CHO; LEA + LCHO, low EA and low CHO intake. The EDE-QS cut-off score is ≥*15; the EAI cut-off is* ≥*24 while scores between 13 and 23 indicate some symptoms and 6 and 12 no symptoms; the MDDI cut-off is* ≥*39*.

Between-group differences were observed for the median EDE-QS score (Kruskal–Wallis H = 11.469, *p* = 0.009), with the LEA + LCHO scoring higher compared to SEA + SCHO (Figure 2). In contrast, EAI and MDDI scores did not differ significantly between groups.

Of the participants, three (7.3%) reached the EDE-QS, five (12.2%) the EAI, and four (9.8%) the MDDI cutoff. Two LEA + LCHO athletes reached the EDE-QS and MDDI cutoffs, and one of them was also considered at risk of compulsive exercise, according to the EAI. Both those athletes had menstrual disturbances. One athlete in the SEA + LCHO group exceeded the EDE-QS cutoff only and had menstrual disturbances. Two athletes (SEA + LCHO and SEA + SCHO) scored above the MDDI cutoff only; of them, one had menstrual disturbances and one was using hormonal contraceptives. Four athletes scored above the EAI cutoff only (LEA + SCHO *n* = 2, SEA + SCHO *n* = 1, SEA + LCHO *n* = 1); of them, one had menstrual disturbances and one was a contraceptive user.

## 4 Discussion

The aim of the present study was to compare dietary intake, nutrition status, and occurrence of REDs (problematic LEA) symptoms between groups of female athletes displaying different patterns of EA and CHO intake in real-life situations. The findings suggest that athletes with patterns of LEA and LCHO are at greater risk of developing REDs than the other three groups.

Moreover, low CHO intakes were often accompanied by low intakes of other macro- and micronutrients that are essential for exercise capacity, training adaptation, and overall health.

### 4.1 Within-group characteristics

Approximately 60% of participants in this study came from ball and endurance sports, with the latter sport group often referred to as being at high-risk of REDs and disordered eating due to pressure to be thin or beliefs that a lower body weight leads to better performances (4, 51). Interestingly, 70% of the endurance athletes had both sufficient to optimal EA and CHO, compared to one-third of the ball sport athletes. Moreover, 50% of the athletes with LEA + LCHO patterns were ball sport athletes. The lower number of athletes from the three other sport groups challenge further investigation of sport-specific risk. Yet our findings support that occurrence of REDs is not limited to certain types of sports; moreover, that individual characteristics and various external factors appear to have a bigger impact than type or nature of the training *per se*. What eventually dictates the onset of REDs is insufficient energy intake in relation to training demands (4, 52). Indeed, the training characteristics of participants varied substantially, with the highest number of training hours reported by aesthetic athletes and the lowest by ball and weight-class athletes. Therefore, it appears that the relatively high occurrence of LEA + LCHO in the ball and power sports is not explained by greater training demands or EEE, but rather reasons such as unawareness of energy/nutrition requirements or dietary restrictions (53). In comparison, five out of nine LEA + SCHO athletes were from aesthetic and endurance sports where EEE is often very high. Accordingly, Melin et al. (54) reported a 7-day average EEE of 1,222 kcal and total EE of 3,266 kcal in endurance athletes displaying LEA. Moreover, Brown et al. (55) reported a 7-day average total EE of ∼2,800 kcal and EA of 26 kcal/kg FFM in pre-professional female dancers.

Nutrition periodisation, characterised by adaptable LEA and CHO intakes tailored to the demands of training, is a common practice in endurance and other sports (20, 21). While athletes were not asked if they periodised their nutrition or were following a specific diet, such approaches were likely adhered to by some of the LEA + SCHO athletes. Athletes with high EEE and long training days may also have prioritised CHO intake, while challenged by limited eating opportunities and/or inability to fulfil total energy requirements (51).

Body composition, primarily body fat percentage, differed between the EA + CHO groups. More specifically, the SEA + LCHO group had higher body fat levels compared to the LEA + SCHO and SEA + SCHO groups but not the LEA + LCHO group. However, the cross-sectional nature of this study limits the possibility to investigate potential causal relationships of this finding with REDs. Sport-specific training adaptations and physiological demands, genetics and a plethora of individual factors influence adiposity (56). Theoretically, the observed group differences in body fat levels could be rooted in energy conservation, including reduction in energy metabolism in response to prolonged or repeated LEA and/or LCHO exposure (57). In contrast, no differences were seen for RMR and whole-body BMD.

### 4.2 Energy availability and dietary intake

This study built on the assumption that more frequent exposure to LEA and LCHO could better predict the risk of problematic LEA compared to calculated averages alone (22).

For all participants, the average CHO intake was marginally higher than the 3.0 g/kg BW cutoff used for the categorisation but varied considerably between days for many. Indeed, CHO requirements are subject to change based on training demands and it is of great importance for athletes to be aware of it and ensure that CHO intake is sufficient for the work required (58). Low or restricted intakes of CHO also increase the likelihood of insufficient intakes of other essential nutrients as well. Nutrients work in synergy to support metabolism and other body functions, and any modifications of athletes’ diet must therefore be well considered to avoid nutrient inadequacies or deficiencies (19). Current sport-specific nutrition recommendations for protein are 1.2–2.0 g/kg BW (59, 60). The average protein intake of all athletes in the two SCHO groups exceeded 1.0 g/kg BW while there were a few cases with average intakes <1.0 g/kg BW in both LCHO groups. Thus, exposure to LEA and LCHO appears to modulate protein intake, which may consequentially result in missed opportunities for recovery and training adaptations (59, 60). No group differences were found in energy and macronutrient intakes from sport foods and supplements, which suggests that such products were often used to compensate low dietary intakes or seen as a convenient solution. In accordance, poor diet is among potential reasons for using supplements, with ∼30% of track and field athletes reporting this reason (61). Moreover, dietary restrictions seemed to be primarily focused on food sources but not sport foods and supplements, perhaps due to beliefs that the latter is healthier and/or provides athletic advantages (31). In agreement with the literature (62), vitamin D and iron intakes were below the recommended intakes for the majority of all athletes, and this points towards important room for improvement in favour of bone health and wellbeing.

### 4.3 Nutrition status

The IOC has listed adequate vitamin D status (>30 ng/mL/∼80 nmol/L) as critical, especially for athletes at risk and/or those recovering from REDs, for the sake of bone health and reduced risk of bone stress injuries (4). The role of vitamin D in skeletal muscle function and sport performance has also been highlighted (63). Of all participants, only 25% reached adequate intake levels for athletes. Moreover, half of the LEA + LCHO athletes had vitamin D insufficiency or deficiency (<50 nmol/L) compared to none of SEA + SCHO. Measurements in this study were conducted at one time point between April and September, with majority of the females in the larger research project measured in the spring/early summer (April–June). In Iceland and the other Nordic countries, it is recommended to supplement vitamin D, especially in the wintertime (40). It is thus possible that those who took vitamin D supplements in the wintertime had recently stopped or taken a break from supplementation in the springtime. That could indeed explain why those who currently took vitamin D supplements did not have higher serum concentrations compared to those who did not report current use. Moreover, seasonal variation in vitamin D status among elite athletes has been reported in the literature, with highest levels measured in the late summer but lowest in late winter (64). Therefore, and given that many barely exceeded 50 nmol/L, it is possible that the incidence of deficiency for vitamin D would have been different if measurements had been taken at other seasons. Accordingly, major determinants of vitamin D status are sun exposure, supplementation and regular intake of foods high in vitamin D (40). Although between EA + CHO group differences were not significant, low levels in the LEA + LCHO group spark a special worry in terms of long-term bone health. Moreover, based on the presented data, many of the participating athletes would benefit from yearlong vitamin D supplementation.

It has been suggested that suboptimal iron status can be either a cause or consequence of REDs, although this remains to be supported by more robust scientific evidence (65).

The present study found no group differences for any of the iron markers. The design of this study, and the fact that it includes females with variable menstrual function, does not allow for deep evaluation of the interrelations between iron status and occurrence of REDs. However, the findings point out that potential relationships (or lack thereof) of iron metabolism with REDs are likely complicated by menstrual characteristics and/or use of contraceptives in females. Accordingly, low iron and ferritin levels were predominantly found in normally menstruating females. It has been well established that menstruation is a primary non-exercise-related cause of iron loss in females, and therefore ferritin levels are commonly lower in females than males (66). Moreover, exercise-related factors, such as haemolysis, haematuria, and gastrointestinal bleeding, contribute towards increased blood loss, while elevated post-exercise hepcidin levels potentially impair iron absorption from meals consumed soon after exercise (67). Whether iron status is directly linked to REDs or not, it holds a key role in oxygen transport, fuel utilisation, and other key functions, and is therefore extremely important for athletic performance. Moreover, co-occurrence of problematic LEA and iron deficiency can make a bad situation considerably worse (68).

### 4.4 Self-reported symptoms of LEA

#### 4.4.1 Low Energy Availability Questionnaire

Despite a tendency towards highest total LEAF-Q score in the LEA + LCHO group, no differences were observed in any of the LEAF-Q scores between EA + CHO groups. The most likely explanation of this is that the LEAF-Q was initially designed and validated for use in female endurance athletes (27) and does not account for between-sport differences in injury risk and physiological demands. Moreover, contraceptive use limits the utility of the menstrual subscale (69, 70). The LEAF-Q does, however, allow for determination of menstrual function and use of contraceptives (45), as was done in this study, and appears suitable to define those at low or no risk of REDs (69). The only athlete who reported current amenorrhoea was in the LEA + LCHO group, while the remaining six athletes with menstrual disturbances reported oligomenorrhea. Menstrual disturbances, amenorrhoea especially, are a red flag for REDs in females (4); however, apart from pregnancy and contraceptive use, other possible reasons for disturbances could not be ruled out based on the collected data. We do, however, echo the importance of interpreting LEAF-Q outcomes in relation to sport-specific factors and use of contraceptives (27, 69, 70).

Observed group differences in response to many of the LEAM-Q-derived items indicate that problematic LEA is reflected in self-reported outcomes, such as sleep, recovery, training readiness, and general energy levels. LEA + LCHO athletes rated their sleep and energy levels as worse and were less ready to perform during training sessions than SEA + SCHO athletes. Moreover, both LEA groups rated their recovery as worse compared to the SEA + SCHO group, with large effect sizes. That is in agreement with reports from qualitative investigations based on in-depth interviews with female athletes (71). To the best of our knowledge, this is the first study to evaluate scoring on the LEAM-Q-derived items in relation to EA assessments. In the initial LEAM-Q validation attempt in males (46), scoring on the LEAM-Q was validated against objective physiological measures but not calculated EA. Although further research on the validity of these aspects when screening for REDs in male and female athletes alike is warranted, the presented findings provide insight on potential subjective outcomes to look for when screening for REDs in female athletes.

#### 4.4.2 Disordered eating, compulsive exercise, and muscle dysmorphia

Previously, we have reported associations of self-reported disordered eating, compulsive exercise, and muscle dysmorphia with symptoms of REDs in male and female athletes (26). In accordance, here the LEA + LCHO group scored highest on the EDE-QS, with two out of nine exceeding the questionnaire cutoff. Those two also exceeded the MDDI cutoff, and one of them was also considered at risk of compulsive exercise according to EAI. One athlete in the SEA + LCHO group scored above the EDE-QS cutoff compared to none from the two SCHO groups.

Accordingly, disordered eating and eating disorders are generally considered a special risk factor of REDs (4), and CHO avoidance is among the potential symptoms of disordered eating (72). Although between-group differences were only observed for EDE-QS scores, there are indications that multifactorial body image concerns and disordered eating behaviours are among external modulators of energy availability. Moreover, as outlined previously (26), the drive for thinness and aesthetic physique are not mutually exclusive. Our results are also in agreement with studies reporting that the association of compulsive exercise with REDs is diminished by the absence of disordered or otherwise insufficient eating habits (73).

### 4.5 Limitations

The present study has some limitations. First, due to its explorative nature, it should first and foremost be regarded as a step towards further understanding of the intersection between adaptable and problematic LEA. Therefore, a larger and/or better controlled study (e.g., in terms of sport groups) with other outcomes, such as site-specific bone mineral density (i.e., not only whole body) and the primary REDs indicators recently suggested by the IOC (4, 36), could have resulted in somewhat different or more comprehensive findings. It should be acknowledged that some errors in evaluating dietary intake and energy expenditure are inevitable. However, we strived to limit such errors by using a photo-assisted mobile application that was specially tailored for the convenient reporting of dietary intake and training. Accordingly, it has been suggested that the replacement of traditional approaches with digital applications may reduce chances of dietary intake misreporting and even, importantly, reduce participant burden (74). We had one-on-one discussions with each athlete to ensure they were fully informed about the aims of this reporting, and the importance of not making changes to their routines due to participation in the study. It must also be clearly stated that MET scores do not provide precise individualised estimates of EEE, as they were primarily designed for the standardisation of physical activity intensities (42). Extracting measured but not estimated RMR from total energy expenditure to yield energy cost of exercise alone, as was done for most participants in this study, partly compensates for this limitation (75). Also important is that coding, calculations, and cleansing of nutrition and training data were performed by well-trained experts who each had their separate task in the process. Therefore, any potential errors should apply to estimations for all participants, which allows for reliable comparisons. Seven days is only a snapshot of the individuals’ life and does not provide information on potential variations between weeks, months, or training periods. That, in addition to the study design, does not allow for any conclusions on causality. Finally, selection bias and other considerations for the greater research project have been addressed elsewhere (26).

## 5 Conclusion

The findings suggest that patterns of low energy availability and low carbohydrate intakes increase the risk of REDs in female athletes. Moreover, that athletes displaying such patterns also have insufficient intakes of other macro- and micronutrients to support health and performance. The highest occurrence of apparently intentional causes of problematic LEA, such as dietary restrictions and disordered eating, but not greatest training demands, was observed among LEA + LCHO athletes. Contrarily, some LEA + SCHO cases might predominantly relate to unintentional mismatch between energy intake and high energy expenditure. Larger studies, powered to identify true statistical and clinically important differences and including evaluation of primary indicators of problematic LEA, are needed to confirm the findings. While occasional LEA and LCHO exposure is unlikely to be harmful and can potentially stimulate training adaptations, repeated exposures to LEA and LCHO should be avoided as they are associated with a cluster of negative implications in female athletes.

## Data Availability

The data relevant to this study is included in the article and/or supplemental material. Requests to access the datasets should be directed to the corresponding author: biva@hi.is.
