# Supplementary material for: Patterns of energy availability and carbohydrate intake differentiate between adaptable and problematic low energy availability in female athletes
Source: Front Sports Act Living. 2024 May 9;6:1390558. doi: 10.3389/fspor.2024.1390558 (PMC11111999; doi:10.3389/fspor.2024.1390558)
Supplement: Supplementary file 1 [file Datasheet1.pdf]

Supplemental file

Frontiers in Sports and Active Living – Sport and Exercise Nutrition; "Patterns of energy availability and carbohydrate intake differentiate between adaptable and problematic low energy availability in female athletes"; Birna Vardardottir (biva@hi.is), Sigridur Lara Gudmundsdottir, Ellen Alma Tryggvadottir, Anna S. Olafsdottir; University of Iceland, Faculty of Health Promotion, Sport & Leisure Studies, Reykjavik, Iceland.

The figures below show A) patterns of energy availability (EA), energy intake (EI), and exercise energy expenditure (EEE) in kcal/kg FFM), and B) relative macronutrient intake for all participants (n=41). Figures are arranged by EA+CHO groups but their numbers are otherwise random (and different from participant IDs). The registration days (numbers) are shown on the x-axis. Dotted line on the energy availability figures represents the established LEA cut-off (<30 kcal/kg FFM), and dotted line on the macronutrient figures represent the cut-off used for low CHO intakes (3 g/kg BW). Information about sport group and age are provided below the figures.

SEA+SCHO (Sufficient to optimal energy availability + sufficient to optimal carbohydrate intake)

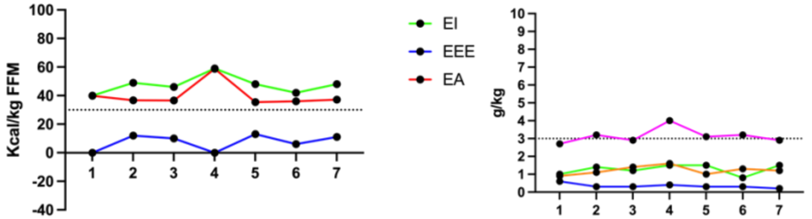

Athlete 1, weight-class, 22 y.

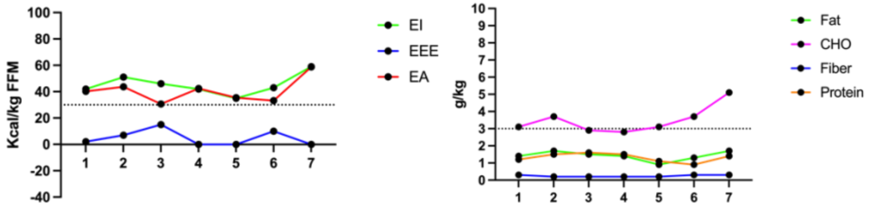

Athlete 2, ball, 23 y.

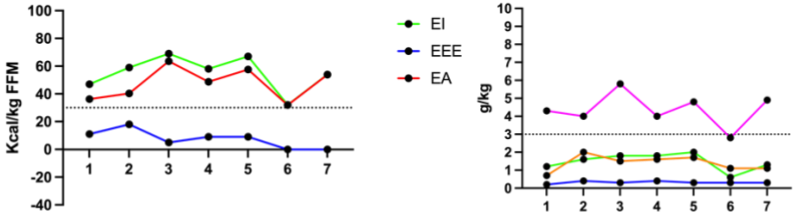

Athlete 3, aesthetic, 16 y.

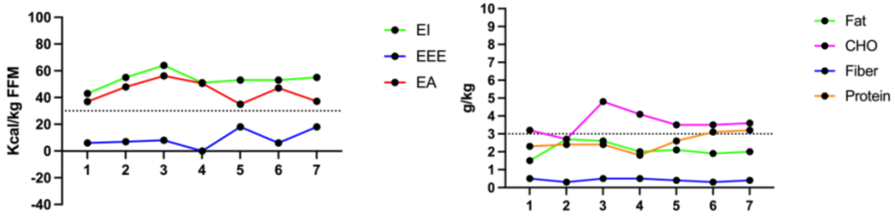

Athlete 4, endurance, 27 y.

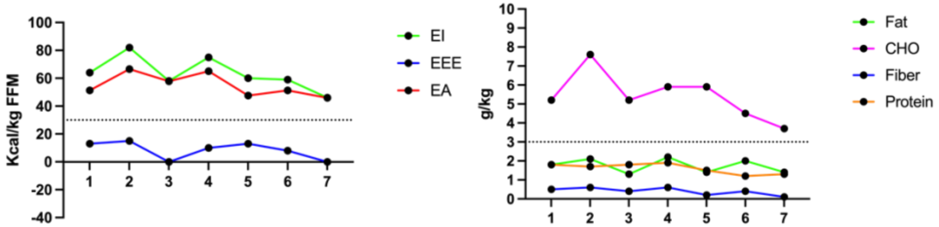

Athlete 5, endurance, 16 y.

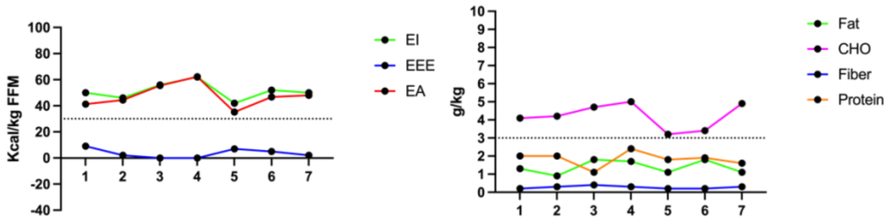

Athlete 6, weight-class, 17 y.

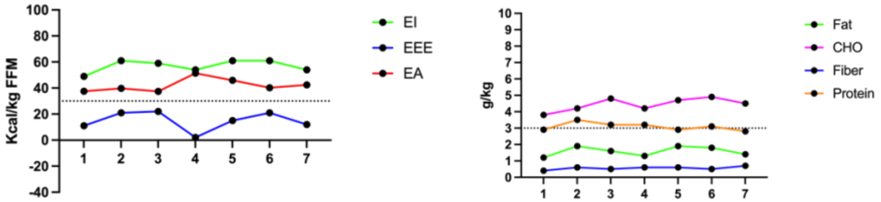

Athlete 7, endurance, 18 y.

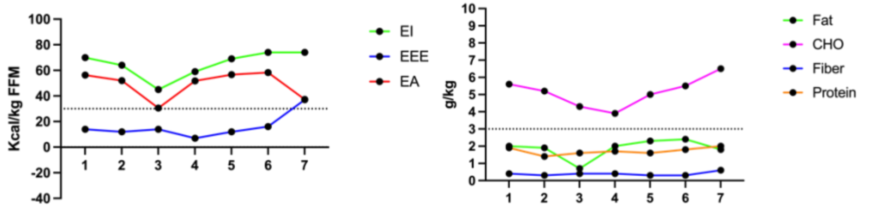

Athlete 8, endurance, 35 y.

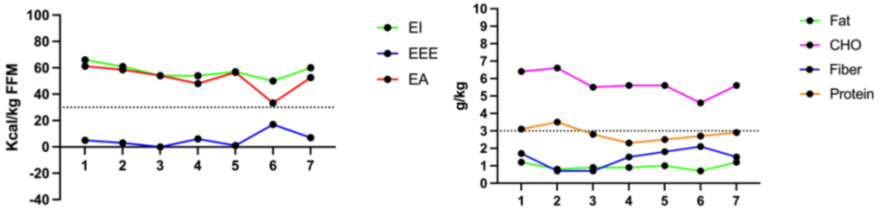

Athlete 9, ball, 19 y.

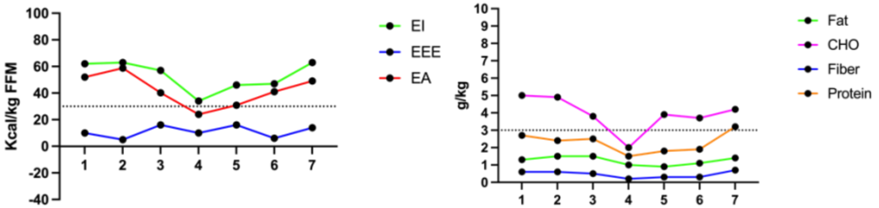

Athlete 10, ball, 18 y.

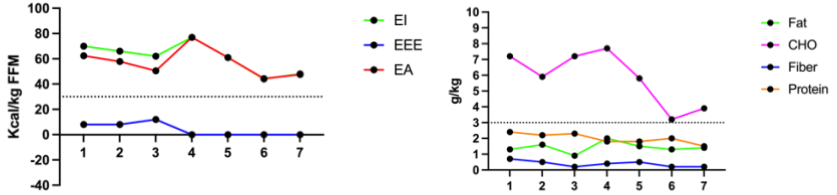

Athlete 11, ball, 15 y.

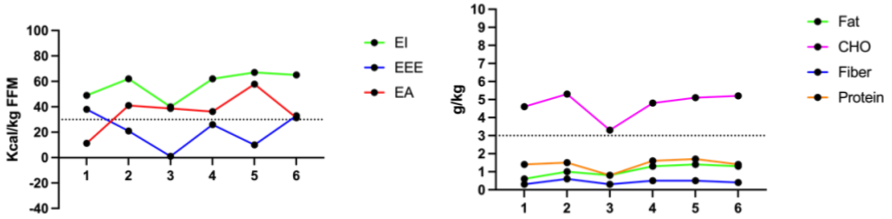

Athlete 12, endurance, 31 y.

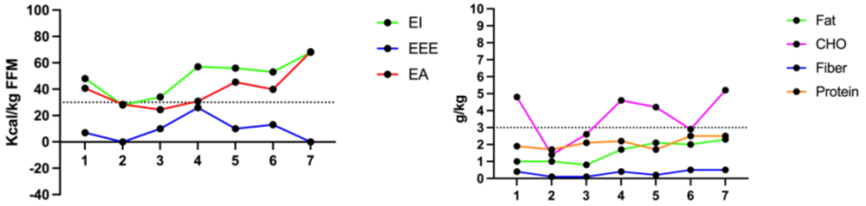

Athlete 13, endurance, 33 y.

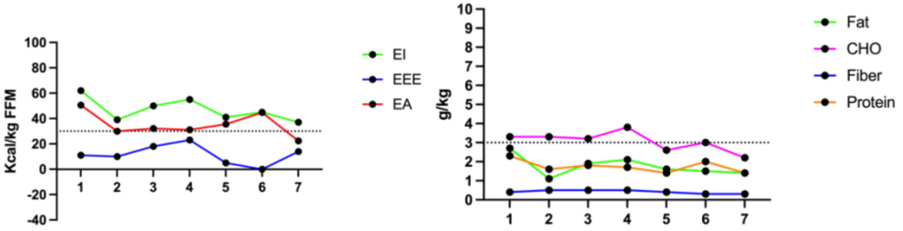

Athlete 14, ball, 30 y.

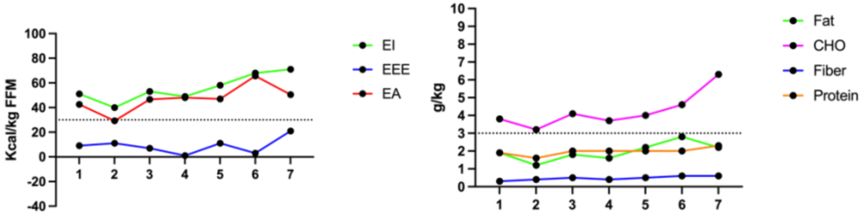

Athlete 15, endurance, 49 y.

SEA+LCHO (sufficient to optimal energy availability + low carbohydrate intake)

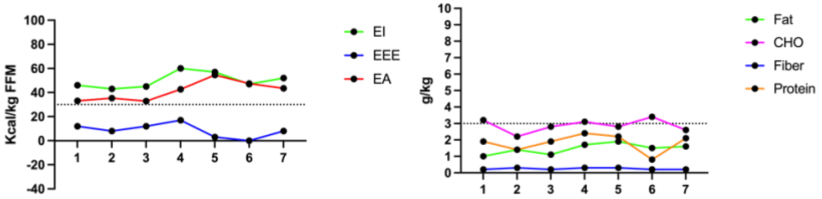

Athlete 16, aesthetic, 19 y.

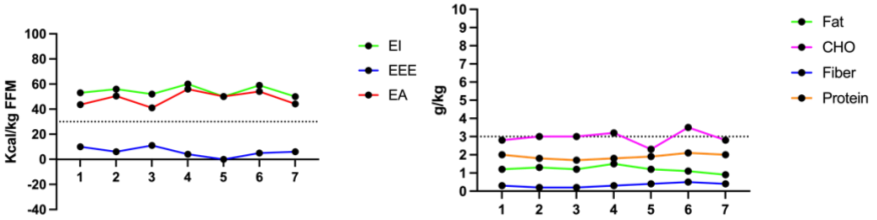

Athlete 17, weight-class, 23 y.

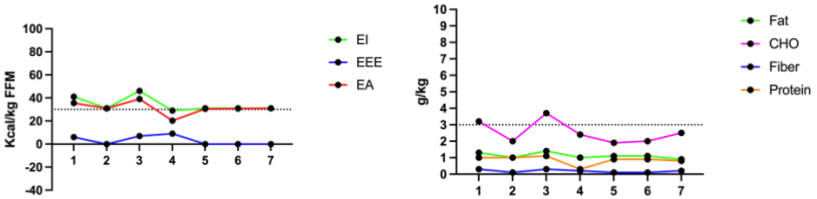

Athlete 18, ball, 16 y.

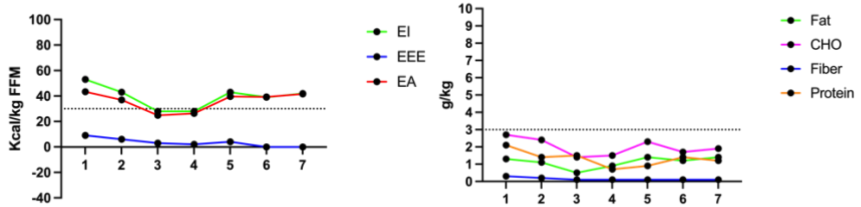

Athlete 19, ball, 25 y.

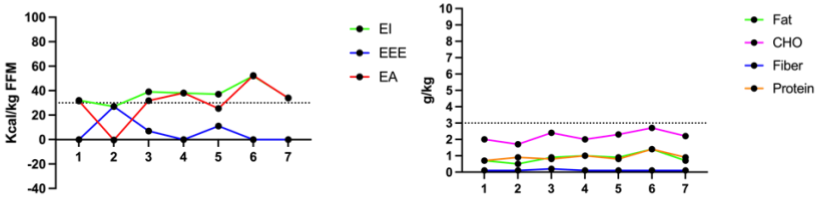

Athlete 20, ball, 20 y.

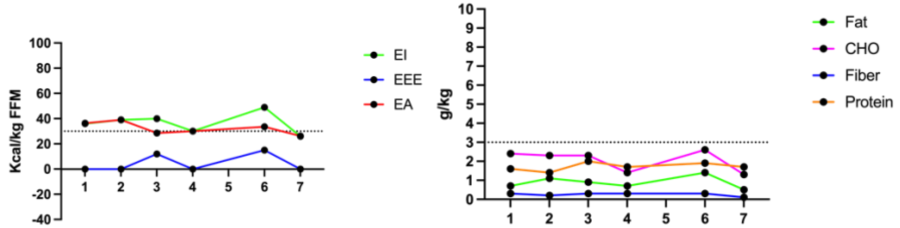

Athlete 21, weight-class, 22 y.

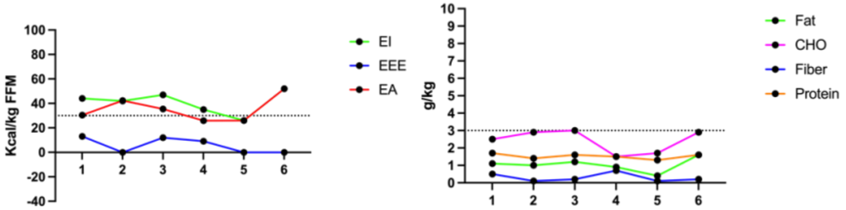

Athlete 22, power, 18 y.

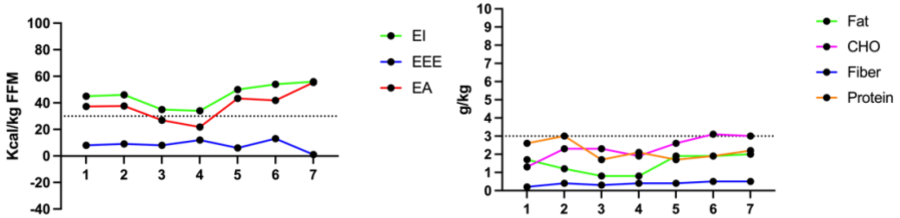

Athlete 23, aesthetic, 18 y.

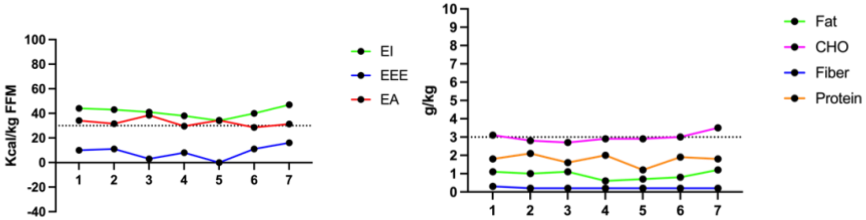

Athlete 24, weight-class, 27 y.

LEA+SCHO (Low energy availability + sufficient to optimal carbohydrate intake)

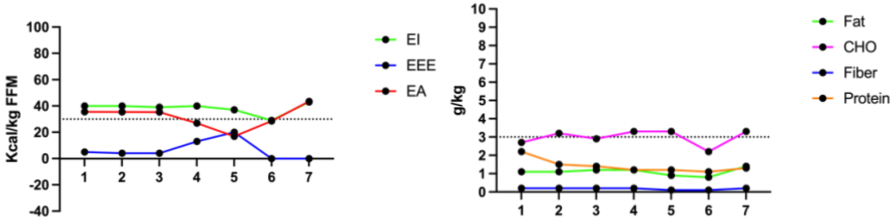

Athlete 25, ball, 28 y.

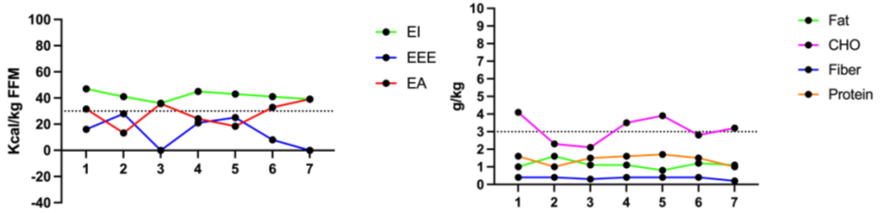

Athlete 26, aesthetic, 18 y.

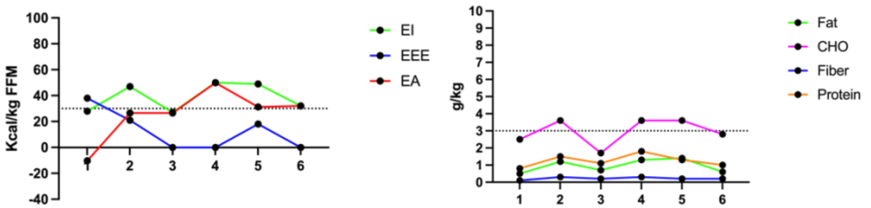

Athlete 27, aesthetic, 17 y.

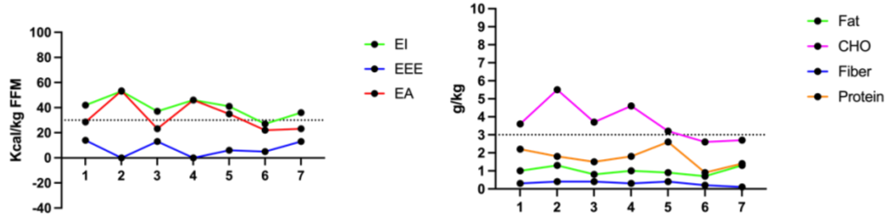

Athlete 28, ball, 28 y.

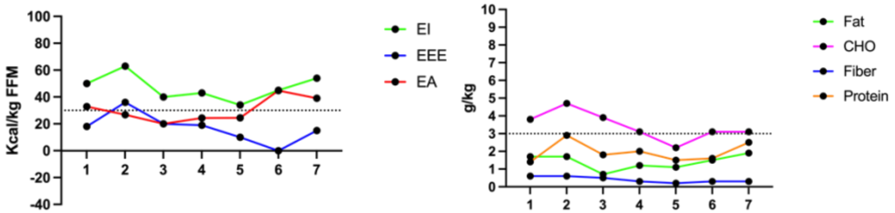

Athlete 29, ball, 22 y.

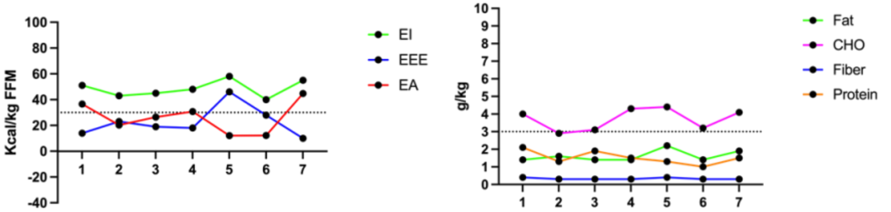

Athlete 30, aesthetic, 17 y.

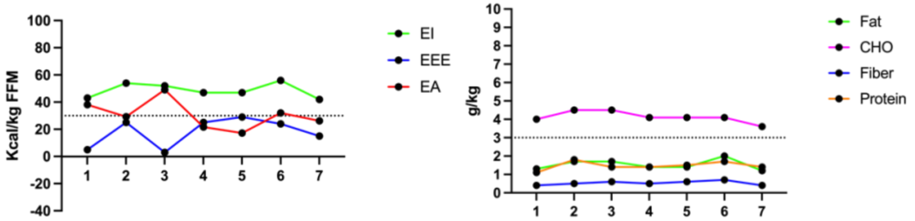

Athlete 31, endurance, 20 y.

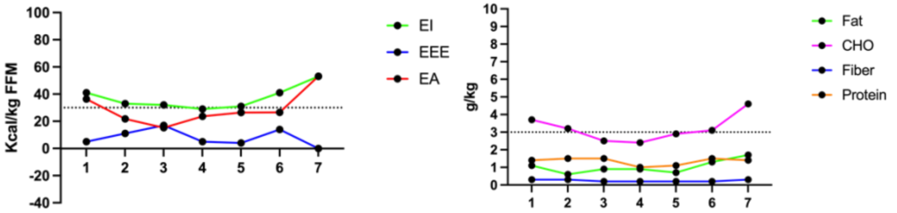

Athlete 32, endurance, 21 y.

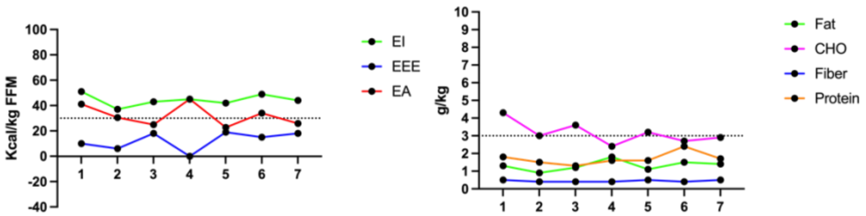

Athlete 33, ball, 19 y.

LEA+LCHO (Low energy availability + low carbohydrate intake)

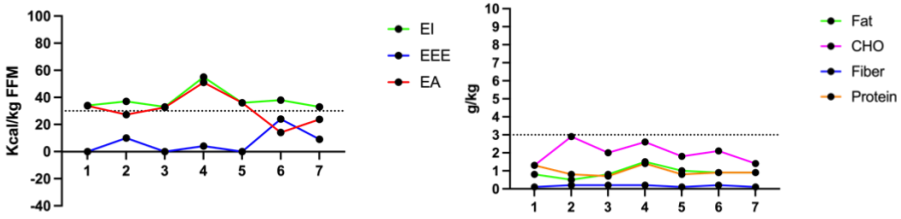

Athlete 34, ball, 24 y.,

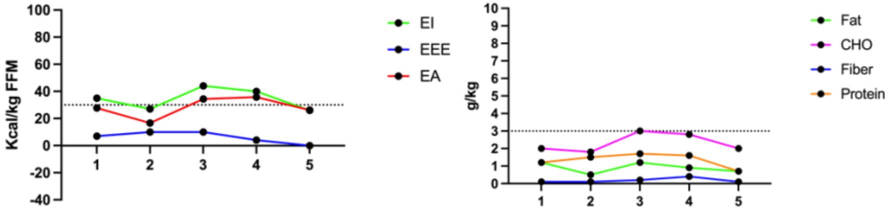

Athlete 35, ball, 39 y.

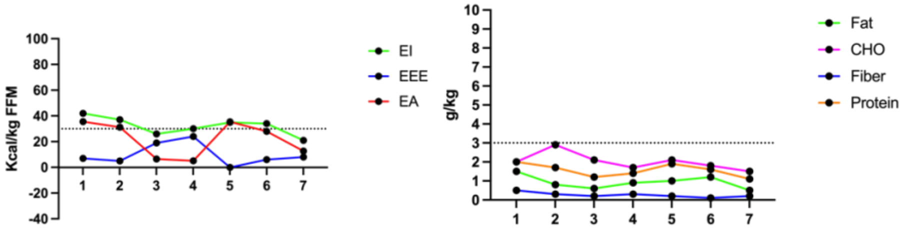

Athlete 36, endurance, 26 y.

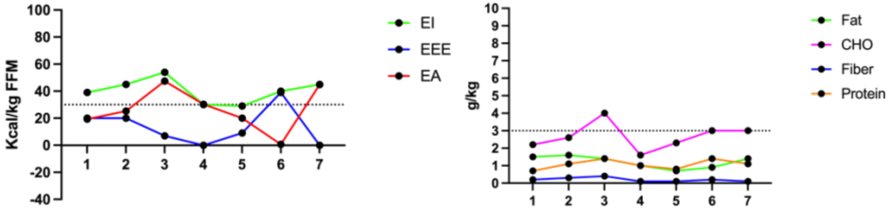

Athlete 37, ball, 16 y.

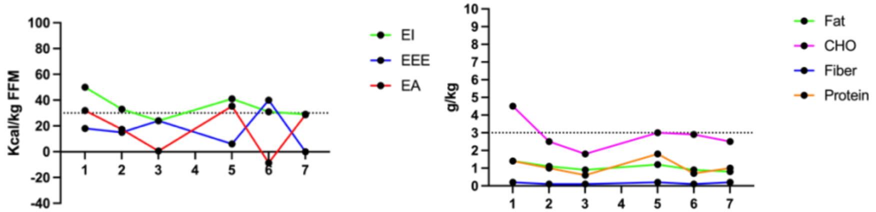

Athlete 38, power, 20 y.

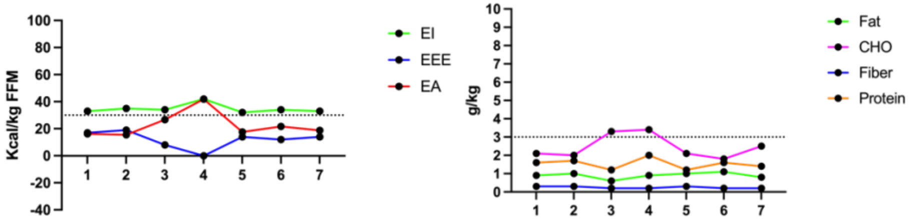

Athlete 39, ball, 20 y.

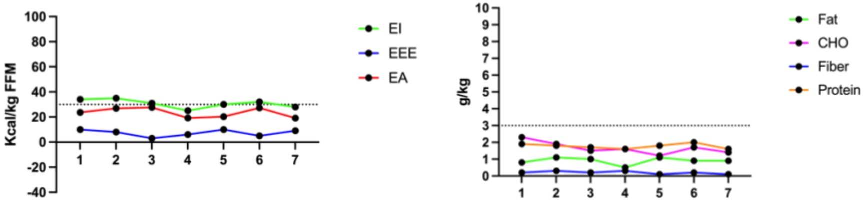

Athlete 40, power, 33 y.

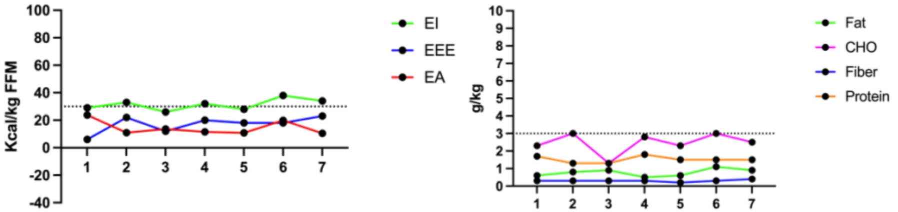

Athlete 41, aesthetic, 20 y.
